# Supplementary material for: Factors associated with willingness to take COVID-19 vaccine among pregnant women at Gondar town, Northwest Ethiopia: A multicenter institution-based cross-sectional study
Source: PLoS One. 2022 Nov 3;17(11):e0276763. doi: 10.1371/journal.pone.0276763 (PMC9632816; doi:10.1371/journal.pone.0276763)
Supplement: S1 File — (PDF) [file pone.0276763.s001.pdf]

English version questionnaires

Part I: Socio-demographic data of the study participants

No	Variable	Possible Answers
100	Age in years	__________
101	Place of resident	A. Urban B. Rural
102	Marital status	A. Married B. Single<br>C. Divorced D. Widowed
103	Educational status	A. Not attended formal education<br>B. Primary education<br>C. Secondary and above
104	Occupation	A. Housewife B. Employed C. Merchant<br>D. Unemployed E. Student F. Daily laborer
105	Average monthly income	______________ Ethiopian Birr

Part II:-Maternal and health related questions

No	Variable	Category
200	Do have any contact with COVID-19 patient?	A. Yes B. No
201	Is there any member of household diagnosed with COVID-19?	A. Yes B. No
202	Do you have any relatives who have been diagnosed with COVID-19?	A. Yes B. No
203	Have you friends that have been diagnosed with COVID-19?	A. Yes B. No
204	Did have tested for COVID-19?	A. Yes B. No
205	If yes for Q no. 204, what was the result?	A. Positive B. Negative
206	Number of pregnancy	A. Primigravida<br>B. Multigravida
207	Have you history of abortion or still birth	A. Yes B. No
208	No of alive children	_____________
209	Did have ANC visit?	A. Yes B. No

210	If yes for Q 210, what is the number of ANC Visits?	A. 1 time<br>B. 2 times<br>C. 3 times<br>D. ≥ 4 times
211	Have you any known maternal chronic medical illness?	A. Yes B. No
212	If yes for Q 211, what type of chronic medical illness do you have?	A. Hypertension<br>B. Diabetes mellitus<br>C. Kidney problems<br>D. Others (specify…)

Part III:-COVID 19 vaccine acceptance related questionnaires

No	Variable	Category
300	Did you have any information about COVID-19 vaccine?	A. Yes B. No
301	If yes for Q 300, source of information	A. Media<br>B. Health professional<br>C. Kebele leaders<br>D. Religious leaders<br>E. Other (specify)
302	Did you get COVID-19 vaccination previously?	A. Yes B. No
303	Do you have an intention to accept the COVID-19 vaccine?	A. Yes B. No
304	Are you concerned about your unborn baby getting COVID-19?	A. Yes B. No

Part IV: - COVID-19 vaccine knowledge questions

Instruction: These are ten knowledge statements about COVID-19 vaccine and each statement has two possible answers. Read each item carefully and encircle your answers.

No	Questions/statements	Categories	
401	Do you think avoiding overcrowding can decrease risk of COVID-19	Yes	No
402	Is COVID-19 caused by virus?	Yes	No
403	Reinfection can occur with COVID 19 disease?	Yes	No
404	If a pregnant woman gets COVID-19,she is more likely to have severe illness	Yes	No
405	Fever is a sign and symptom of corona virus disease?	Yes	No
406	Dry cough is a sign and symptom of corona virus disease?	Yes	No
407	Difficulty of breathing is a symptom of corona virus disease?	Yes	No
408	People with pre-existing disorders have weak prognostic result if infected with corona virus?	Yes	No
409	Staying indoors and using face mask can prevent transmission of corona virus disease?	Yes	No
410	Without developing signs and symptoms, individuals with corona virus disease can spread the virus to others?	Yes	No

Part V: - COVID-19 vaccine attitude questions

Instruction: There are about ten attitude statements about COVID-19 vaccine and each statement has five possible answers on a five-point scale. Read each item carefully and circle:

1= If you **strongly disagreed** about the statement.

2= If you **disagreed** about the statement.

3= If you **neither agreed nor disagreed** (neutral) about the statement.

4= If you **agreed** about the statement.

5= If you **strongly agreed** about the statement.

No	Questions/ statements	1	2	3	4	5
501	If a pregnant woman gets COVID-19, the illness could harm her unborn baby					
502	COVID-19 vaccine can cause a person to get sick					
503	Giving vaccine to a pregnant woman will benefit her fetus and baby					
504	Getting vaccine during pregnancy benefits for the pregnant woman					
505	If family members recommended vaccine, I would get vaccinated					
506	May you encourage your family/friends/relatives to get vaccinated					
507	It is not possible to reduce the incidence of COVID-19 without vaccination					
508	COVID-19 infection is due to our sin and wrong doings					
509	COVID-19 attacks only white people					
510	COVID-19 is common only in old people					
