# Supplementary material for: Factors associated with willingness to take COVID-19 vaccine among pregnant women at Gondar town, Northwest Ethiopia: A multicenter institution-based cross-sectional study
Source: PLoS One. 2022 Nov 3;17(11):e0276763. doi: 10.1371/journal.pone.0276763 (PMC9632816; doi:10.1371/journal.pone.0276763)
Supplement: Attachment — Submitted filename: Response to Reviewers.docx [file pone.0276763.s004.docx]

**Date፡ June 21, 2022**

**Rebuttal letter**

**Dear Editor,**

Thank you for allowing us the opportunity to submit a revised draft of the manuscript “Factors Associated With Willingness to Take COVID-19 Vaccine among Pregnant Women at Gondar Town, Northwest Ethiopia: A Multicenter Institution-Based Cross-Sectional Study” for publication in the Journal of PLOS ONE. We appreciate the time and effort that you and the reviewers devoted to providing feedback on our manuscript. All are valuable and insightful comments that led to possible improvements in the current version. All authors took part in the revision process, and we addressed all the raised issues by the reviewers.

Please check the blue below for a point-by-point response to the editor/reviewers. All page numbers relate to the tracked changes version of the document. In the manuscript, all changes have been highlighted in red.

Best regards,

Zewdu Bishaw

[Zest7@yahoo.com](mailto:Zest7@yahoo.com)

Lecturer, at Department of Nursing, Injibara University

**A point-by-point response to the editor and/or reviewers**

Thank you for submitting your manuscript to PLOS ONE. After careful consideration, we feel that it has merit but does not fully meet PLOS ONE’s publication criteria as it currently stands. Therefore, we invite you to submit a revised version of the manuscript that addresses the points raised during the review process.

**Editor's comments:**

**Editor's comment:** My understanding that “a lottery method” is a “random selection”. While “lottery method” might be understandable term, but not widely used. I would suggest using “random selection” instead.

**Authors’ response:** Thanks for your kind reminders. Based on the suggestion, we used the term “random selection” instead of “a lottery method”. See the track change document on page 5, line numbers 131 to 132 and 137.

**Editor's comment:** Make sure the manuscript meets the PLOS One journal requirements in style and format.

**Authors’ response:** Thank you! We act in accordance with the general PLOS ONE journal requirements in style and format. For instance, we summited figures and references based on the journal requirements. Take a look at the whole document.

**Editor's comment:** It looks like the authors used a systematic random selection approach when recruiting participants. What are the limitations of such approach?
**Authors’ response:** Thanks for your inquiry. Among many more, a few limitations are:

- Each participant has an equal or known chance of being picked in simple random sampling. Systematic sampling, on the other hand, picks a participant for each predetermined interval.
- Although systematic sampling takes less time than random sampling, it might lead to skewed results if the data set contains patterns.

**Editor's comment:** Who are the language experts? Bilingual translators?

**Authors’ response:** Thank you for your inquiry. Here, we're talking to mention Amharic and English professionals /academic instructors. We replaced “language experts” with “language professionals”. See the track change document on page 7, line number 161.

**Editor's comment:** More information is needed to understand reliability and validity of the questionnaire in the study population. Was the questionnaire pre-tested? If yes, how many participants responded? Any modifications were introduced? How experts assessed the validity of the items? What approach was undertaken to perform the expert review? Have the authors assess internal validity of the questionnaire? Have the authors performed confirmatory or exploratory factor analysis to assess interrelationships of items? Have the authors used any methods to validate the questionnaire? Have the authors accounted for the complex survey design in all statistical analyses? Without proper accounting for survey design, estimates and corresponding confidence intervals might be incorrectly calculated.

**Authors’ response:** Thanks for your suggestions and concerns. All are constructive.

We modified using the following statement “the reliability of the questionnaire was checked using the reliability index measurement (Cronbach's alpha), which was 0.87 and its content validity was assessed by public health specialists”. “The questionnaire was pretested on 53 study participants (10% of the sample size) with similar demographic characteristics. Accordingly, some language and typographic corrections have been addressed as needed”. See the track change document on page 7, lines 163 to 165, and 181 to 183.

Please submit your revised manuscript by Jul 20 2022 11:59PM. If you will need more time than this to complete your revisions, please reply to this message or contact the journal office at [plosone@plos.org](mailto:plosone@plos.org). When you're ready to submit your revision, log on to https://www.editorialmanager.com/pone/ and select the 'Submissions Needing Revision' folder to locate your manuscript file.

Please include the following items when submitting your revised manuscript:

- A rebuttal letter that responds to each point raised by the academic editor and reviewer(s). You should upload this letter as a separate file labeled 'Response to Reviewers'.
- A marked-up copy of your manuscript that highlights changes made to the original version. You should upload this as a separate file labeled 'Revised Manuscript with Track Changes'.
- An unmarked version of your revised paper without tracked changes. You should upload this as a separate file labeled 'Manuscript'.

If you would like to make changes to your financial disclosure, please include your updated statement in your cover letter. Guidelines for resubmitting your figure files are available below the reviewer comments at the end of this letter.

If applicable, we recommend that you deposit your laboratory protocols in protocols.io to enhance the reproducibility of your results. Protocols.io assigns your protocol its own identifier (DOI) so that it can be cited independently in the future. For instructions see: <https://journals.plos.org/plosone/s/submission-guidelines#loc-laboratory-protocols>. Additionally, PLOS ONE offers an option for publishing peer-reviewed Lab Protocol articles, which describe protocols hosted on protocols.io. Read more information on sharing protocols at <https://plos.org/protocols?utm_medium=editorial-email&utm_source=authorletters&utm_campaign=protocols>.

We look forward to receiving your revised manuscript.

Kind regards,

Alpamys Issanov
Academic Editor
PLOS ONE

**Journal Requirements:**

**When submitting your revision, we need you to address these additional requirements.**

1. Please ensure that your manuscript meets PLOS ONE's style requirements, including those for file naming. The PLOS ONE style templates can be found at https://journals.plos.org/plosone/s/file?id=wjVg/PLOSOne_formatting_sample_main_body.pdf and 
https://journals.plos.org/plosone/s/file?id=ba62/PLOSOne_formatting_sample_title_authors_affiliations.pdf

**Authors’ response:** Accepted. We comply with general PLOS ONE journal requirements in style and format.

2. We suggest you thoroughly copyedit your manuscript for language usage, spelling, and grammar. If you do not know anyone who can help you do this, you may wish to consider employing a professional scientific editing service.

Whilst you may use any professional scientific editing service of your choice, PLOS has partnered with both American Journal Experts (AJE) and Editage to provide discounted services to PLOS authors. Both organizations have experience helping authors meet PLOS guidelines and can provide language editing, translation, manuscript formatting, and figure formatting to ensure your manuscript meets our submission guidelines. To take advantage of our partnership with AJE, visit the AJE website (http://learn.aje.com/plos/) for a 15% discount off AJE services. To take advantage of our partnership with Editage, visit the Editage website (www.editage.com) and enter referral code PLOSEDIT for a 15% discount off Editage services.  If the PLOS editorial team finds any language issues in text that either AJE or Editage has edited, the service provider will re-edit the text for free.

Upon resubmission, please provide the following:
 
The name of the colleague or the details of the professional service that edited your manuscript

A copy of your manuscript showing your changes by either highlighting them or using track changes (uploaded as a *supporting information* file)

A clean copy of the edited manuscript (uploaded as the new *manuscript* file)”

Authors’ response: Thank you very much for pointing this out. All authors went through the entire manuscript to eliminate language usage, spelling, and grammar mistakes. Additionally, we use free online grammar checker tools to address these concerns like:

- Free Grammar Checker | Grammarly (<https://www.grammarly.com/grammar-check>),
- Ginger Spell and Grammar Checker for Windows (<https://ginger-spell-and-grammar-checker.en.softonic.com/>),
- Becorrect English Grammar & Spell Checker (<https://becorrect.com/new/rbmajixihbteqocf>) and
- Free Grammar Checker | QuillBot (<https://quillbot.com/grammar-check>)

3. We note that you have stated that you will provide repository information for your data at acceptance. Should your manuscript be accepted for publication, we will hold it until you provide the relevant accession numbers or DOIs necessary to access your data. If you wish to make changes to your Data Availability statement, please describe these changes in your cover letter and we will update your Data Availability statement to reflect the information you provide.

**Authors’ response:** Thank you. We made changes to our Data Availability statement and described these changes in our cover letter.

4. We note that you have indicated that data from this study are available upon request. PLOS only allows data to be available upon request if there are legal or ethical restrictions on sharing data publicly. For information on unacceptable data access restrictions, please see http://journals.plos.org/plosone/s/data-availability#loc-unacceptable-data-access-restrictions.

In your revised cover letter, please address the following prompts:

a) If there are ethical or legal restrictions on sharing a de-identified data set, please explain them in detail (e.g., data contain potentially identifying or sensitive patient information) and who has imposed them (e.g., an ethics committee). Please also provide contact information for a data access committee, ethics committee, or other institutional body to which data requests may be sent.

b) If there are no restrictions, please upload the minimal anonymized data set necessary to replicate your study findings as either Supporting Information files or to a stable, public repository and provide us with the relevant URLs, DOIs, or accession numbers. Please see http://www.bmj.com/content/340/bmj.c181.long for guidelines on how to de-identify and prepare clinical data for publication. For a list of acceptable repositories, please see http://journals.plos.org/plosone/s/data-availability#loc-recommended-repositories.

We will update your Data Availability statement on your behalf to reflect the information you provide

Authors ‘response: Agree. There are no ethical or legal restrictions on sharing our data set. As a result, the minimal set of anonymized data has been uploaded as supporting information files. Our Data Availability Statement was updated, and we explained the changes in our cover letter.

**Reviewers' comments:

Reviewer's Responses to Questions**

**Comments to the Author**

1. Is the manuscript technically sound, and do the data support the conclusions?
The manuscript must describe a technically sound piece of scientific research with data that supports the conclusions. Experiments must have been conducted rigorously, with appropriate controls, replication, and sample sizes. The conclusions must be drawn appropriately based on the data presented.

Reviewer #1: Yes

Reviewer #2: Yes

2. Has the statistical analysis been performed appropriately and rigorously?

Reviewer #1: Yes

Reviewer #2: Yes

3. Have the authors made all data underlying the findings in their manuscript fully available?

The [PLOS Data policy](http://www.plosone.org/static/policies.action#sharing) requires authors to make all data underlying the findings described in their manuscript fully available without restriction, with rare exception (please refer to the Data Availability Statement in the manuscript PDF file). The data should be provided as part of the manuscript or its supporting information, or deposited to a public repository. For example, in addition to summary statistics, the data points behind means, medians and variance measures should be available. If there are restrictions on publicly sharing data—e.g. participant privacy or use of data from a third party—those must be specified.

Reviewer #1: Yes

Reviewer #2: Yes

4. Is the manuscript presented in an intelligible fashion and written in standard English?

PLOS ONE does not copyedit accepted manuscripts, so the language in submitted articles must be clear, correct, and unambiguous. Any typographical or grammatical errors should be corrected at revision, so please note any specific errors here.

Reviewer #1: Yes

Reviewer #2: Yes

5. Review Comments to the Author

Please use the space provided to explain your answers to the questions above. You may also include additional comments for the author, including concerns about dual publication, research ethics, or publication ethics. (Please upload your review as an attachment if it exceeds 20,000 characters)

**Reviewer #1:** I read the manuscript entitled "" by ??? et al with great interest and would like to congratulate the authors for their effort. However, there are some minor points to improve the quality of manuscript:

**Reviewer’s comment 1)** in introduction authors discuss previous reports of vaccination COVID among pregnant women as follow: "The refusal to accept the COVID-19 vaccine is estimated to be high and greatly differs between countries (19). For example, an online survey of pregnant women in 16 countries found that COVID-19 vaccine acceptance was more than 80% in Mexico and India, but lower than 45% in the United States, Australia, and Russia (20). In Switzerland (7), the United Kingdom (21), China 4 (22), and Ethiopia (23) the willingness to accept the COVID-19 vaccine among pregnant women was found 62.1%, 29.7%, 77.4%, and 70.7% respectively (7, 21, 22). The main factors that determine an individual’s intent to take the COVID-19 vaccine include gender, age, educational status, occupational status, income, pre-existence of chronic disease, attitude towards and knowledge of COVID-19 (23-28). Moreover, multiple myths and conspiracy theories on vaccines and COVID-19 (5, 29, 30) would also potentially affect the COVID-19 vaccine acceptance." Please add the recent meta-analysis published evaluating the global acceptance rate of covid-19 vaccine among pregnant women. PMID: 34670322 DOI: 10.1055/a-1674-6120

**Authors’ response:** Thank you for bringing this to our attention! Based on the suggestion given, we incorporated a recent meta-analysis estimating the global acceptance rate of the covid-19 vaccination among pregnant women. See in the track change document on page 4, line number 90 up to 92.

**Reviewer’s comment 2)** Page7 (methods) How about the history of TdP or flu vaccine in pregnancy and the chance of accepting COVID-19 vaccination?

**Authors’ response:** Thank you! Although receiving influenza vaccination during the previous season was linked to a greater likelihood of COVID-19 vaccine acceptance, we didn't look at this link.
**Reviewer’s comment 3)** Page 8 (methods) move ethics to the beginning of the methods.
**Authors’ response:** We value the feedback provided by the reviewer. However, based on the general style and format guidelines of the PLOS ONE publication, we kindly believe that not moving ethics to the start of the methods section does not influence the work's reputation.
**Reviewer’s comment 4)** Page 13 and 14. it is not clear for which variables the adjustment model has been performed?

**Authors’ response:** Thank you for requesting further clarification. First, all variables were entered into the bivariable model. Then variables having a p-value of ≤ 0.2 and or those whose CI did not include 1 were entered into the adjustment (multivariable) model. For detail, we kindly invite you to look at the data processing and analysis part of the material and methods section.

**Reviewer #2:** Authors performed an interesting cross-sectional study in Northwest Ethiopia, exploring the acceptance rate of covid-19 vaccine among pregnant women. They observed a low rate of acceptance, and found that women would get it when there is enough knowledge and attitude towards the vaccine. Indeed, this is the proof that when people tend to get information, the fear of unknown and unproven consequences lowers, with the effect of favoring higher uptake of vaccines. Therefore, governments should push on informative politics and campaigns with reinforcement of positive news about vaccines against SARS-CoV-2 as recent literature is showing.

The study is quite well conducted and reported. However, here are some remarks:
**Reviewer’s comment:-** there are no line numbers and this makes difficult to refer to certain sentence. When authors will resubmit the manuscript, please use line numbers

**Authors’ response:** We agreed with the concerns and have incorporated them in the revised work.

**Reviewer’s comment:** - please reformulate the methods section in the abstract which although quite understandable is not very clearly written and difficult to read especially in relation to statistical analysis, as well as the corresponding section of methods in the manuscript

**Authors’ response:** Accepted and rewritten to make clear. See the track change document on page 2, lines number 32 to 42.

**Reviewer’s comment:** - The discussion needs to be implemented: first of all, authors referred to various surveys conducted through the world, but missed to mention the first two performed in Italy, which was one of the first country that faced the diffusion of the infection after China (Carbone L, Mappa I, Sirico A, Di Girolamo R, Saccone G, Di Mascio D, Donadono V, Cuomo L, Gabrielli O, Migliorini S, Luviso M, D’antonio F, Rizzo G, Maruotti GM. Pregnant women perspectives on SARS-COV-2 vaccine. Am J Obstet Gynecol MFM. 2021 Mar 23:100352. Doi: 10.1016/j.ajogmf.2021.100352. Epub ahead of print. ----- Mappa I, Luviso M, Distefano FA, Carbone L, Maruotti GM, Rizzo G. Women perception of SARS-CoV-2 vaccination during pregnancy and subsequent maternal anxiety: a prospective observational study. J Matern Fetal Neonatal Med. 2021 Apr 11:1-4. doi: 10.1080/14767058.2021.1910672. Epub ahead of print.)

**Authors’ response:** Thank you for bringing our attention to this. Accepted and incorporated into the revised document. See the track change document on page 15, line numbers 279 up to 281.

**Reviewer’s comment:** - In addition, a systematic review found more or less a similar percentage of uptake, and well discuss mechanism for the implementation of vaccine uptake (Shamshirsaz AA, Hessami K, Morain S, Afshar Y, Nassr AA, Arian SE, Asl NM, Aagaard K. Intention to Receive COVID-19 Vaccine during Pregnancy: A Systematic Review and Meta-analysis. Am J Perinatol. 2021 Oct 20. doi: 10.1055/a-1674-6120. Epub ahead of print. PMID: 34670322.)

**Authors’ response:** Thank you for pointing this out. Agreed and included. See the track change document on page 4, line numbers 90 up to 92 and page 15, line number 280.

**Reviewer’s comment:** - in this regard, in the last part of the discussion, authors could focus on how local policies could be implemented according to local healthcare issues and pregnancy management facilities

**Corrections:**
**Introduction part**

**Reviewer’s comment:** Introduction page 3, “in the movement”: please reformulate

**Authors’ response:** Thank you. We revised it. See in the track change document on page 3, line number 65.

**Reviewer’s comment:** Introduction page 3, “cesarian” should be “cesarean”

**Authors’ response:** Thank you. The word “cesarian” is replaced with “cesarean”. See in the track change document on page 3, line number 70.

**Reviewer’s comment:** introduction page 3, “such the above-mentioned” could be “the abovementioned”

**Authors’ response:** Accepted and corrected! See in the track change document on page 3, line number 74.

**Reviewer’s comment:** introduction page 3, “particularly in pregnancy continues….” Please reformulate

**Authors’ response:** Accepted and reworked! See in the track change document on page 3, line number 84.

**Reviewer’s comment:** please delete the ORCID URL placed in page 5, in the middle of the methods, before figure 1

**Authors’ response:** Thank you. We deleted the “ORCID URL placed in page 5’’. See in the track change document on page 5, line number 141.

**Material and methods** **part**

**Reviewer’s comment:** Data collection instrument and Study variables, page 7, “by reviewing different literature” could more properly be “after exhaustive literature search”

**Authors’ response:** Thank you! We replaced the phrase “by reviewing different literature” with “after exhaustive literature search”. See in the track change document on page 7, line number 158.

**Reviewer’s comment:** Data collection instrument and Study variables, page 7, “it was the outcome variable of the study” should be “it represents the main outcome of the study”

**Authors’ response:** Accepted and revised as “it represents the main outcome of the study”. See in the track change document on page 7, line numbers 173 to 174.

**Reviewer’s comment:** discussion, page 15, I suggest to not use the term “perfect solution” when referring to the vaccine against SARS-CoV-2. Indeed, is a very useful and protective tool which has demonstrated to be effective in reducing the risk of contracting the infection but also reducing the severity of the eventual illness. However, “perfect solution” seems too much

**Authors’ response:** Thank you for your genuine review. We changed “perfect solution” to “ideal solution”. See in the track change document on page 15, line number 273.

**Reviewer’s comment:** please report the references consistently among them and as per journal guidelines

**Authors’ response:** Thank you for pointing this out. We reported all references as per journal guidelines i.e. in the text, all the reference numbers are cited in square brackets.
