# Supplementary material for: Factors associated with willingness to take COVID-19 vaccine among pregnant women at Gondar town, Northwest Ethiopia: A multicenter institution-based cross-sectional study
Source: PLoS One. 2022 Nov 3;17(11):e0276763. doi: 10.1371/journal.pone.0276763 (PMC9632816; doi:10.1371/journal.pone.0276763)
Supplement: Attachment — Submitted filename: Response to Reviewers.docx [file pone.0276763.s005.docx]

**Date: September 22, 2022**

**Rebuttal letter**

**Alpamys Issanov,**

**Academic Editor,**

**PLOS ONE**

**Dear Academic Editor Dr. Issanov**

**Subject:** Submission of revised paper titled “Factors Associated with Willingness to Take COVID-19 Vaccine among Pregnant Women at Gondar Town, Northwest Ethiopia: A Multicenter Institution-Based Cross-Sectional Study.”

Thank you for your email dated August 9, 2022 enclosing the editor and or reviewers' comments. We appreciate the time and effort that you and the reviewers devoted to providing feedback on our manuscript. All are valuable and insightful comments that led to possible improvements in the current version. All authors took part in the revision process. We have carefully reviewed the comments and have revised the manuscript accordingly. Our responses are given in a point-by-point manner below. The blue text below shows the point-by-point responses to the editor/reviewers. All page numbers relate to the tracked changes version of the document, and each revision has been highlighted in red.

Best regards,

Zewdu Bishaw

[Zest7@yahoo.com](mailto:Zest7@yahoo.com)

Lecturer, Department of Nursing, Injibara University

**A point-by-point response to the editor and/or reviewers**

Thank you for submitting your manuscript to PLOS ONE. After careful consideration, we feel that it has merit but does not fully meet PLOS ONE’s publication criteria as it currently stands. Therefore, we invite you to submit a revised version of the manuscript that addresses the points raised during the review process.

**Editor's comments:**

1. I would recommend expanding on this statement “Third, the nature of the study design, being cross-sectional, does not show the exact connection between cause and effect.” Why do the authors believe that it is challenging to determine causality?

**Authors’ response:** Thank you! We tried to modify the statement. .” See the track change document on page 16, line numbers 336 up to 337.

But, the reason why we authors believe that it is challenging to determine causality, was based on the following scientific justifications **(Solem, 2015)**;

- Even though a cross-sectional study was not a clinical trial, there is a study design limitation because the associations of exposure and outcomes are simultaneously measured. Thus, it is impossible to assess any temporal relationships between exposures and outcomes in cross-sectional studies.
- Cross-sectional study is a one-time measurement of results and it is difficult to determine a cause-and-effect relationship from the outcome.
- It is difficult to assess cause and effect relationships in a cross-sectional study. This is because the cross-sectional study is a single-time analysis of the exposure and the result.

2. Please add limitations of the systematic random sampling in the limitations section.

**Authors’ response:** As suggested by the editor, we have added limitations of systematic random sampling in the limitation section which stated as “Fourth, although systematic random sampling takes less time than random sampling, it might lead to skewed results if the data set contains patterns. Hence, the results of our study need to be interpreted with caution.” See the track change document on page 16, line numbers 337-340.

3. Numbers in the tables can be rounded to one or two decimal places.

Authors’ response: Thank you for your recommendation. We rounded table 4 to two decimal places. See the track change document on pages 11, 12 and 13, line number 262-264.

4. What was the conceptual framework for examining the research question? I do see that the independent variables were selected based on the threshold of p<0.2. But have the authors considered epidemiologically, conceptually important variables to be included in the model? Or was the analysis purely explorative/descriptive? I would encourage to include a visual representation of the theoretical framework of the study so a reader can relate to it.

**Authors’ response:** Thank you! As suggested, we have added the conceptual framework to the revised manuscript. See the track change document on page 4, line number 90 and 92-93.

5. When the authors stated that “[variables]…were entered into the multivariable analysis to control for possible confounders”, it seems a bit counterintuitive to the research question. The study aims to find factors associated with the vaccine acceptance, while adjusting for confounding factors usually is done in the causative research question (one primary exposure variable). I would recommend aligning the research question with the selected analysis.

**Authors’ response:** While we appreciate the reviewer’s comment, we respectfully have different view. As numerous experts have pointed out, confounding hides the true impact of a variable. Therefore, confounding must be addressed at the stages of research design and analysis. At design stage, it can be addressed by using randomization, restriction, or matching. Multivariate analysis is yet another popular method for addressing confounding ***(Jager et al., 2008).*** As of our experiences, we have seen a large amount of scholars using a cutoff value for independent variables to enter into the multivariable analysis. They usually use 0.2 or 0.25 as a cut of point to take variables from bivariable to multivariable analysis. Again, study which titled “Health care workers intention to accept COVID-19 vaccine and associated factors in southwestern Ethiopia, 2021” was published in PLOS ONE journal previously using 0.25 as a cutoff value ***(Angelo et al., 2021).*** Hence, we think it doesn’t significantly disturb the research question.

6. I also noticed that confidence intervals for some independent variables were extremely large. For example, for age 35+. Have the authors attempted to perform sensitivity analysis with different categorization of age?

**Authors’ response:** We appreciate your inquiry. We made an effort to carry out sensitivity analysis using different age categories. For instance, we first divided ages into 5 groups: 18–24, 25–30, 31–35, 36–40, and >40 years. Based on this category, the Chi-Square test assumption that stated "Expected value of cells should be 5 or more in at least 80% of cells" was broken since the bulk (72.4%) of our participants were found in the age categories of 25-34. To allay this issue, we categorized age as stated in the manuscript. See the track change document on page 11, table 4.

7. While I appreciate the authors adding information on how the survey tool was developed, I am still concerned about the validity and reliability of the tool. For example, the authors stated that the reliability of the tool was tested using the Cronbach’s alpha which was equal to 0.87. However, my understanding is that there are several constructs in the questionnaire and one reliability measurement is not enough.

**Authors’ response:** We think this is an excellent suggestion. We have reworked as “the reliability of the tool was checked using Cronbach's alpha coefficient, the values were 0.87 for the overall tool, 0.73 for attitude subscale, and 0.82 for knowledge subscale.” See the track change document on page 6, line numbers 149-152.

8. I am also curious how the final 10 questions for vaccine knowledge and attitude were derived? What were the initial questions and what were the number of questions? How were they selected?

**Authors’ response:** Thanks for your inquisitiveness. Questions for vaccine knowledge and attitude were prepared after an exhaustive literature search *(Mose and Yeshaneh, 2021, Kasemy et al., 2020, Wang et al., 2020)*. To measure vaccine knowledge, 10 knowledge questions were asked. Each question had 2 possible answers: 1 for “no” and 2 for “yes.” Participants who correctly answered 80% or more of the knowledge questions were considered as having “good knowledge” otherwise “poor knowledge.” In the same way, participant's vaccine attitude was also measured using 10 attitude questions. Each question had a 5-point Likert scale (1 was assigned for strongly disagree, 2 for disagree, 3 for neutral, 4 for agree, and 5 for strongly agree). Subsequently, participants who responded to more than or equal 80% (≥80%) of the attitude-related questions were categorized as having “favorable attitude” if not as having “unfavorable attitude.” See the track change document on page 6, line numbers 154-167. Additionally, the questions on vaccine knowledge and attitude were included in the revised manuscript as a supporting document.

9. Have the authors considered different thresholds when selecting for attitude and vaccine knowledge constructs? What were the results?

**Authors’ response:** Thanks you! During the analysis, different thresholds/criteria were taken into account for attitude questions. For example, we intended to combine "strongly disagree", “disagree” and "neutral" responses together in one category and responses "agree" and "strongly agree" in another category. However, for a flawless understanding of the result and because of prior evidence classification criteria *(Mose and Yeshaneh, 2021),* the participant's attitude score was classified as favorable if it was equal or over 80% and unfavorable if it was below 80% as it stands in this manuscript. But for honestly speaking, we didn’t consider different thresholds for vaccine knowledge questions. Because there was textual support, which states that participants who scored more than or equal to 80% of knowledge assessment questions, were reported as good knowledge otherwise poor *(Mose and Yeshaneh, 2021, Kasemy et al., 2020).*

10. In the analytical part, have the authors considered potential multicollinearity in the analysis? Some of the constructs may have overlapping areas that could cause collinearity. Whether this concern could have affected the results?

**Authors’ response:** Thanks for your genuine concerns! To see the correlation between independent variables, multicollinearity test was carried out using variance inflation factor (VIF) and found 2.56. This VIF value confirmed the absence of significant collinearity among independent variables. See the track change document on page 7, line numbers 184 to 187.

11. Please include the questionnaire as a supporting document.

**Authors’ response:** Thanks for your kind reminders. Based on the suggestion given, the questionnaires were included as a supporting document in the revised manuscript. See the track change document on page 16, line numbers 354 to 356.

**References**

ANGELO, A. T., ALEMAYEHU, D. S. & DACHEW, A. M. 2021. Health care workers intention to accept COVID-19 vaccine and associated factors in southwestern Ethiopia, 2021. *PloS one,* 16**,** e0257109.

JAGER, K., ZOCCALI, C., MACLEOD, A. & DEKKER, F. 2008. Confounding: what it is and how to deal with it. *Kidney international,* 73**,** 256-260.

KASEMY, Z. A., BAHBAH, W. A., ZEWAIN, S. K., HAGGAG, M. G., ALKALASH, S. H., ZAHRAN, E. & DESOUKY, D. E. 2020. Knowledge, attitude and practice toward COVID-19 among Egyptians. *Journal of Epidemiology and Global Health,* 10**,** 378.

MOSE, A. & YESHANEH, A. 2021. COVID-19 Vaccine Acceptance and Its Associated Factors Among Pregnant Women Attending Antenatal Care Clinic in Southwest Ethiopia: Institutional-Based Cross-Sectional Study. *International Journal of General Medicine,* 14**,** 2385.

SOLEM, R. C. 2015. Limitation of a cross-sectional study. *American Journal of Orthodontics and Dentofacial Orthopedics,* 148**,** 205.

WANG, J., JING, R., LAI, X., ZHANG, H., LYU, Y., KNOLL, M. D. & FANG, H. 2020. Acceptance of COVID-19 Vaccination during the COVID-19 Pandemic in China. *Vaccines,* 8**,** 482.
